# Whole-genome sequencing of *Beauveria bassiana* KNU-101 using the hybrid assembly approach

**DOI:** 10.1128/mra.00681-23

**Published:** 2024-01-18

**Authors:** GyuDae Lee, Jerald Conrad Ibal, Tae-Hyung Park, Min-Ji Kim, Seung-Dae Choi, Jae-Ho Shin

**Affiliations:** 1 Department of Applied Biosciences, Kyungpook National University, Daegu, Republic of Korea; 2 NGS Core Facility, Kyungpook National University, Daegu, Republic of Korea; 3 Department of Integrative Biotechnology, Kyungpook National University, Daegu, Republic of Korea; University of Maryland School of Medicine, Baltimore, Maryland, USA

**Keywords:** *Beauveria bassiana*, whole-genome sequencing, hybrid assembly, nanopore sequencing, MGI sequencing

## Abstract

In this report, we present the whole-genome sequences of *Beauveria bassiana* KNU-101, a widely recognized entomopathogenic fungus used as a biopesticide. The genome was assembled using a hybrid assembly approach, resulting in 13 scaffolds with a total size of 35,638,224 bp.

## ANNOUNCEMENT

Biopesticides have become increasingly popular for environmentally friendly and safe pest management in integrated crop management over recent decades (1). *Beauveria bassiana* is a known entomopathogenic fungus. However, there are only 25 genome sequences in the National Center for Biotechnology Information (NCBI), including one that is complete (GCA_014607475.1). In this study, we isolated *B. bassiana* KNU-101 from an apple farm located in Yeongju-si, Gyeongsangbuk-do, South Korea (36°52′36.8″N, 128°29′25.2″E). Deceased insects on the leaves were gathered, and 1 g of the sample was mixed with 9 mL of 0.85% saline. We inoculated 0.1 mL of this mixture onto Sabouraud dextrose agar and incubated it at 30°C for 5 days to morphologically identify potential *B. bassiana* strains. Internal trascribed spacer (ITS) region sequencing later confirmed the isolate as *B. bassiana* (GenBank: LY821854.1).

For the sequencing of the genome, *B. bassiana* KNU-101 was cultured in potato dextrose broth (Difco, USA) at 25°C, 200 rpm shaking incubator for 5 days. After incubation, the cell pellet was used for DNA extraction using the Wizard Genomic DNA Purification Kit (Promega, USA), following the manufacturer’s protocol. Any shearing or size-selection step was not performed for ONT sequencing. Then, the library was prepared using a ligation sequencing kit (SQK-LSK109, ONT, UK) and sequenced on the MinION device using the flowcell FLO-MIN111 (R10.3, ONT). For short-read sequencing, the library was prepared using DNBSEQ-G400RS High-throughput Sequencing Kit SE400 (MGI Tech, China) following the manufacturer’s instructions and sequenced on the DNBSEQ-G400RS sequencer. All sequencing operations were performed in the KNU NGS Core facility, Daegu, South Korea. Nanopore sequencing through Guppy basecalling (v.6.0.6, default parameter) high accuracy model yielded a total of 1,822,896 reads (9,827,426,196 bp) with an *N*_50_ read length of 13,949 bp (2). Quality control of the ONT reads was performed using NanoStat (v.1.4.0, default parameter), revealing that 81.1% of the reads had a quality score exceeding 10 (3). For DNBSEQ sequencing, we obtained 45,050,358 single-end reads (13,775,947,789 bp) with an *N*_50_ read length of 217 bp from MGI sequencing after applying quality filtering through Trimmomatic (v.0.3.9, default parameter except “SLIDINGWINDOW:50:30 MINLEN:36”) (4).

Initially, we conducted *de novo* assembly using long read sequences acquired from the Nanopore MinION device. A draft genome consisting of 26 contigs was assembled using Flye (v. 2.8.2-b1691, default parameter except “--nano-raw --genome-size 36 m”), which includes error correction step (5). Afterward, polishing contigs using high-quality reads from MGI sequencing was conducted using POLCA (v. 4.0.5, default parameter) (6). As a result of the polishing step, 357 substitution errors and 7,894 insertion/deletion errors were corrected, and the consensus quality value was improved to 36.35. Polished contigs were scaffolded using the reference genome data from the NCBI (*B. bassiana* HN6 strain) through the CSAR (v.1.1, default parameter except “--nuc” option) (7). After scaffolding contigs using reference data, the final genome of *B. bassiana* KNU-101 was assembled to a total size of 35,638,224 bp, including 13 scaffolds. The maximum scaffold length was 10,066,884 bp with a GC content of 49.49%, and details for each scaffold are presented in Table 1.

**TABLE 1:** Summary of scaffold sizes and %GC contents

Scaffold	Length (bp)	%GC	Number of gaps
Scaffold 1	6,348,137	50.35	4
Scaffold 2	169,942	52.79	2
Scaffold 3	6,023,620	49.24	2
Scaffold 4	10,066,884	49.49	1
Scaffold 5	8,715,070	49.35	1
Scaffold 6	405,420	51.55	1
Scaffold 7	28,007	49.62	0
Scaffold 8	1,385,281	51.31	0
Scaffold 9	2,473,629	44.88	0
Scaffold 11	4,563	49.48	0
Scaffold 12	1,117	52.46	0
Scaffold 14	1,172	53.24	0
Scaffold 15	15,382	49.5	0
Total	35,638,224		11

## Data Availability

The ITS region sequence and assembly data of *B. bassiana* KNU-101 have been submitted to National Center for Biotechnology Information (NCBI) GenBank and have been deposited under the accession numbers LY821854.1 and GCA_025380265.1, BioProject number PRJNA874513, BioSample number SAMN30551667, and WGS numbers JAOANY010000001-JAOANY010000013. Additionally, raw reads are available under SRA accession numbers SRR25392973, SRR25392974, SRR25392975, and SRR25392976.
